# Synthesis and crystal structure of *catena*-poly[[hexa­aqua{μ_3_-2-[bis­(carboxyl­atometh­yl)amino]­terephthalato}dicobalt(II)] penta­hydrate] containing water tapes and penta­mers

**DOI:** 10.1107/S2056989021008343

**Published:** 2021-08-20

**Authors:** Jie Ma, Wen-Zhi Zhang, Jie Xiong, Chun-Yan Yan

**Affiliations:** a College of Chemistry, Chemical Engineering and Materials Science, Zaozhuang, University, Zaozhuang, Shandong, 277160, People’s Republic of China

**Keywords:** crystal structure, 2-aminodi­acetic terephthalic acid, hydrogen bonds, water tape, water penta­mer

## Abstract

The title coordination polymer, {[Co_2_(C_12_H_7_NO_8_)(H_2_O)_6_]·5H_2_O}_*n*_, was crystallized at room temperature from an aqueous solution of 2-aminodi­acetic terephthalic acid (H_4_adtp) and cobalt(II) nitrate. The asymmetric unit consists of one adtp^4−^ ligand, one and two half Co^II^ ions, six water ligands coordinated to Co^II^ ions and five uncoordinated water mol­ecules. Two of the cobalt cations lie on centres of inversion and are coordinated in octa­hedral O_2_(OH_2_)_4_ environments, whereas the other adopts a slightly distorted octa­hedral NO_3_(OH_2_)_2_ environment. The crystal structure contains parallel stacked, one-dimensional zigzag chains, {[Co_2_(C_12_H_7_NO_8_)(H_2_O)_6_]}_*n*_, which assemble into a three-dimensional supra­molecular architecture *via* networks of hydrogen bonds involving the coordinated and free water mol­ecules. One-dimensional ‘water tapes’ are formed, containing alternating six-membered and twelve-membered rings of water mol­ecules, together with water penta­mers, in which a central uncoordinated water mol­ecule is hydrogen bonded to two coordinated and two free water mol­ecules in a tetra­hedral arrangement.

## Abstract 2

The title compound consists of parallel stacked chains of composition {[Co_2_(C_12_H_7_NO_8_)(H_2_O)_6_]}_*n*_ in which Co^II^ cations are linked together through 2-aminodi­acetic terephthalate anions. The chains are held together by networks of hydrogen bonds involving both coordinated and free water mol­ecules in water tapes and penta­mers.

## Chemical context   

Water clusters, which are aggregations of water mol­ecules assembled *via* hydrogen bonding, are often observed in organic and organic–inorganic hybrid crystal structures. To date, a number of discrete water clusters of different sizes and conformations have been identified, including tetra­mers (Thakur *et al.*, 2021 ▸; Ahmed *et al.*, 2018 ▸), penta­mers (Ghosh & Bharadwaj, 2006 ▸), hexa­mers (Zhao *et al.*, 2015 ▸; Li *et al.*, 2020 ▸), hepta­mers (He *et al.*, 2012 ▸; Hedayetullah Mir & Vittal, 2008 ▸), octa­mers (Hao *et al.*, 2013 ▸; Wei *et al.*, 2009 ▸; Ghosh & Bharadwaj, 2006 ▸), deca­mers (Mukhopadhyay & Bernal, 2006 ▸), and other higher member clusters (Liu *et al.*, 2018 ▸; Chen *et al.*, 2020 ▸). In addition, examples of infinite water clusters consisting of one-dimensional water chains or ‘tapes’ (Gacki *et al.*, 2020 ▸; Zhao *et al.*, 2019 ▸; Saraei *et al.*, 2019 ▸; Han *et al.*, 2019 ▸; Liu *et al.*, 2020 ▸; Saraei *et al.*, 2018 ▸), two-dimensional water layers (Mei *et al.*, 2016 ▸) and three-dimensional water frameworks (Huang *et al.*, 2007 ▸, 2019 ▸; Wu *et al.*, 2013 ▸) have also been reported recently. Water clusters are often held in the cavities of the host structures as guest mol­ecules, which can enhance the stability of the structure. Water clusters, when hydrogen bonded to the host structures, play a vital role in assembling organic and organic–inorganic complex mol­ecules into three-dimensional architectures (Thakur *et al.*, 2021 ▸; Zia *et al.*, 2020 ▸; Huang *et al.*, 2019 ▸; Liu *et al.*, 2018 ▸). Our work focuses on the construction of metal complexes using semi-rigid multi­carb­oxy­lic acids containing aminodi­acetate moieties, and analysing the affects of weak hydrogen-bonding inter­actions on their supra­molecular assemblies (Ma *et al.*, 2015*a* ▸). We have previously reported the synthesis of two Cu^II^ complexes based on 2-(carb­oxy­phen­yl)-iminodi­acetic acid (H_3_cpida) and 1,10-phenanthroline (phen), and discussed the influence of hydrogen bonding on the resulting structures (Ma *et al.*, 2015*b* ▸). Herein we report the synthesis and structural characterization of a Co^II^ coordination polymer, {[Co_2_(C_12_H_7_NO_8_)(H_2_O)_6_]·5H_2_O}_*n*_ (**I**), based on 2-aminodi­acetic terephthalic acid (H_4_adtp). The hydrogen-bonding inter­actions in (**I**), which result in the formation of one-dimensional water tapes and isolated water penta­mers, are discussed in detail.
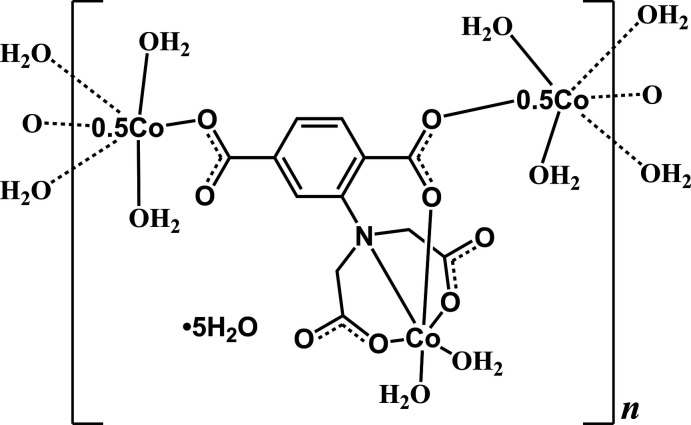


## Structural commentary   

**Figure 1:**
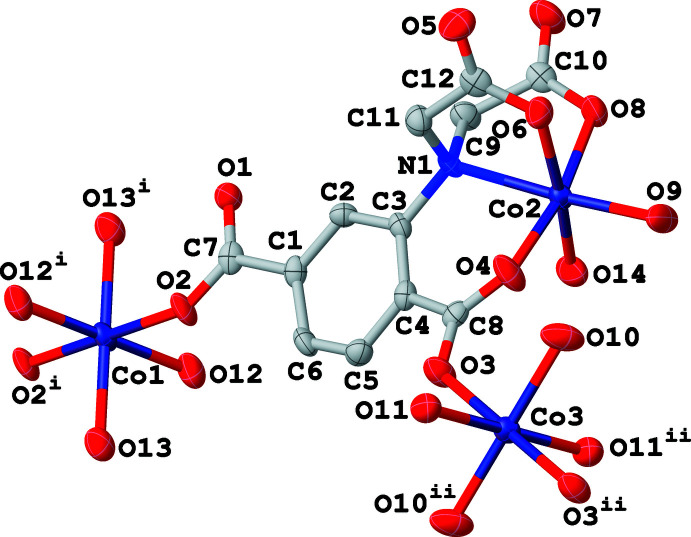
Coordination environments of the Co^II^ ions in (**I**) with displacement ellipsoids shown at the 50% probability level. H atoms have been omitted for clarity. [Symmetry codes: (i) 1 − *x*, −*y*, 2 − *z*; (ii) 1 − *x*, 2 − *y*, 1 − *z*.] Please label C atoms

**Figure 2:**
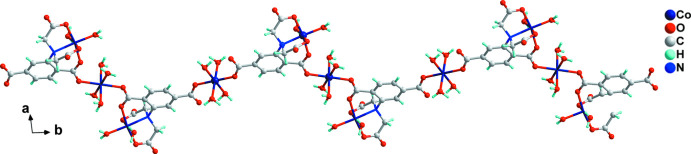
The one-dimensional zigzag coordination chain found in (**I**).

Compound (**I**) crystallizes in the triclinic space group *P*ī. The asymmetric unit comprises three crystallographically distinct Co^II^ ions, one adtp^4−^ ligand, six coordinated water ligands and five free water mol­ecules. Regarding the adtp^4−^ ligand, the carboxyl­ate groups of the aminodi­acetate moiety and that in the *meta-*position adopt monodentate coordination modes on bonding to cobalt, whereas the carboxyl­ate group in the *ortho-*position coordinates in a *syn*–*anti* bidentate bridging fashion (see Scheme). As shown in Fig. 1 ▸, Co2 is located in a distorted octa­hedral N_1_O_5_ environment. The adip^4−^ ion chelates to Co2 *via* the amino nitro­gen atom (N1), two acetate oxygen atoms (O6 and O8) and the *ortho*-position carboxyl­ate oxygen atom (O4). The remaining two *cis*-related sites around Co2 are ligated by oxygen atoms (O9 and O14) of terminal water mol­ecules. Co1 and Co3 both lie on inversion centres and are located in octa­hedral O_6_ environments. In each case, a pair of *trans*-related coordination sites are bonded to equivalent carboxyl­ate oxygen atoms (O2, O2^i^ for Co1; O3, O3^ii^ for Co3). The remaining *trans*-related sites of Co1 and Co3 are ligated by two pairs of equivalent oxygen atoms from terminal water mol­ecules (O12, O12^i^ and O13, O13^i^ for Co1; O10, O10^ii^ and O11, O11^ii^ for Co3, respectively). The length of the Co2—N1 bond is 2.1712 (18) Å and the Co—O distances lie in the range 2.0128 (15)–2.1330 (15) Å, all of which are reasonable values. The adtp^4−^ ligand links the Co1 and Co3 atoms *via* the *ortho*- and *meta-*position carboxyl­ate groups and a zigzag chain is formed by inversion operations with the closest Co1⋯Co3 and Co2⋯Co3 distances being 10.657 (1) and 5.194 (1) Å, respectively (Fig. 2 ▸).

## Supra­molecular features   

**Figure 3:**
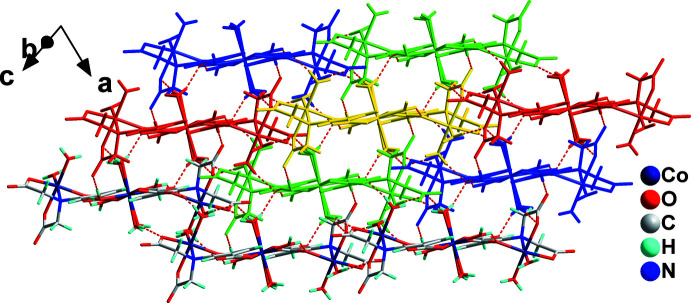
The three-dimensional supra­molecular architecture of (**I**) composed of zigzag coordination chains linked *via* inter­molecular hydrogen bonds shown as dashed red lines. One zigzag chain, highlighted in yellow, associates directly with three pairs of neighbouring chains, which are highlighted in green, red and blue.

**Figure 4:**
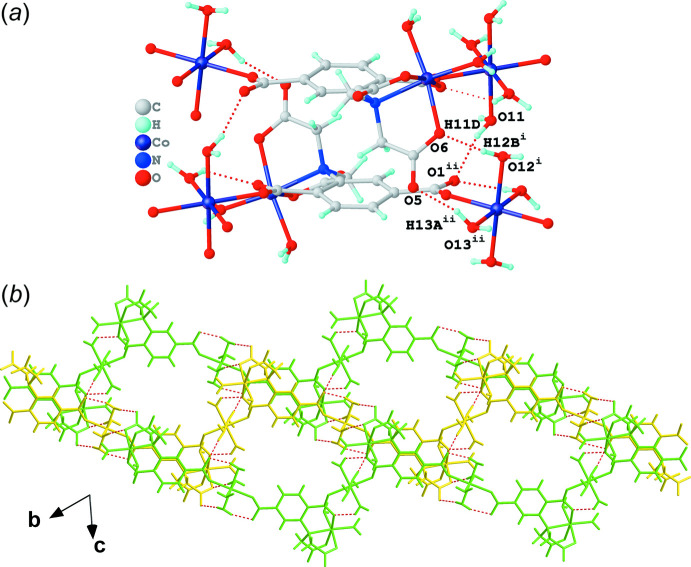
(*a*) Inter­molecular hydrogen bonds of group I shown as red dashed lines and (*b*) the two-dimensional supra­molecular layer generated using the yellow and green chains highlighted in Fig. 3 ▸ linked by the group I hydrogen bonds. [Symmetry codes: (i) *x*, 1 + *y*, −1 + *z*; (ii) 1 − *x*, 1 − *y*, 1 − *z*.]

**Figure 5:**
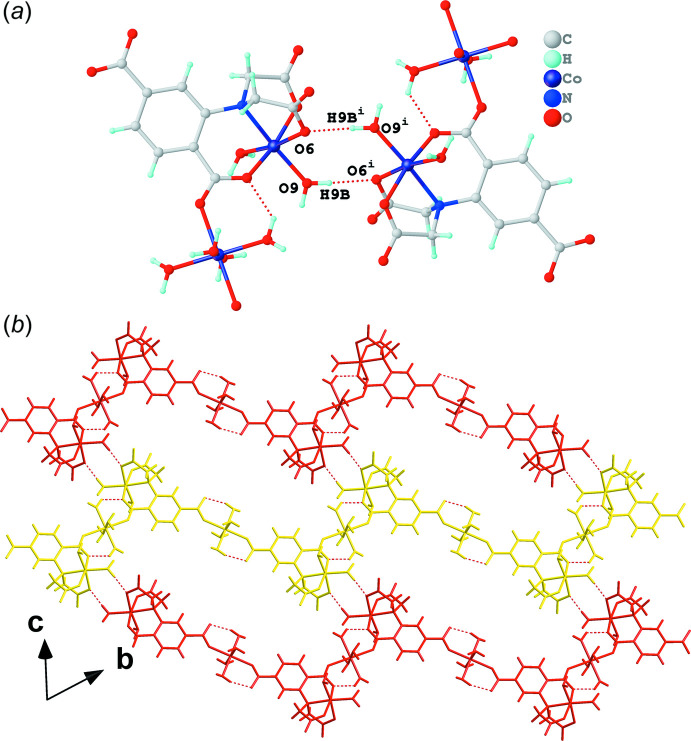
(*a*) Inter­molecular hydrogen bonds of group II shown as red dashed lines and (*b*) the two-dimensional supra­molecular layer generated using the yellow and red chains highlighted in Fig. 3 ▸ linked by the group II hydrogen bonds. [Symmetry code: (i) 2 − *x*, 2 − *y*, −*z*.]

**Figure 6:**
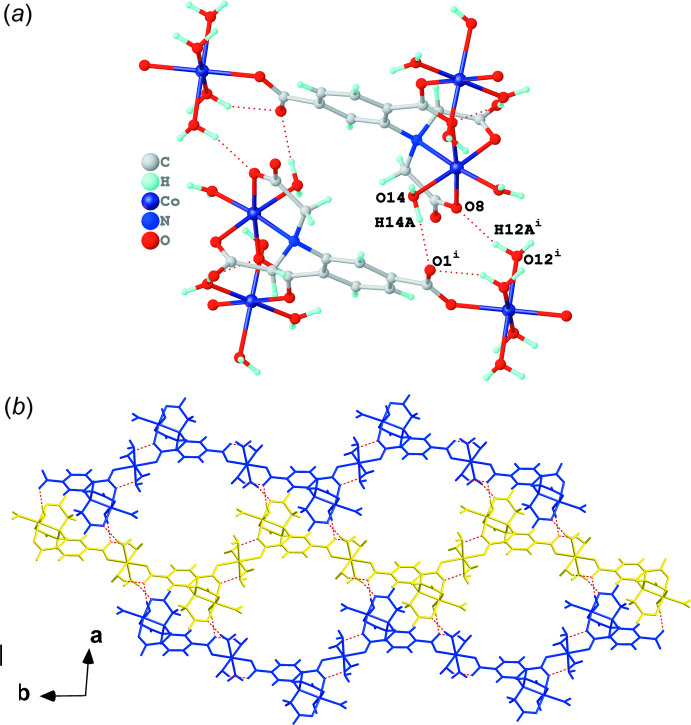
(*a*) Inter­molecular hydrogen bonds of group III shown as red dashed lines and (*b*) the two-dimensional supra­molecular layer generated using the yellow and blue chains highlighted in Fig. 3 ▸ linked by the group III hydrogen bonds. [Symmetry codes: (i) 2 − *x*, 1 − *y*, 1 − *z*.]

The zigzag chains are arranged parallel to each other and inter­molecular hydrogen bonds (Table 1 ▸) between adjacent chains play a significant role in assembling the three-dimensional supra­molecular architecture. As shown in Fig. 3 ▸, one zigzag chain, highlighted in yellow, associates directly *via* hydrogen bonds with three pairs of nearby chains, which are highlighted in green, red and blue. The inter­molecular hydrogen bonds between two adjacent chains can be classified into three groups: (I) inter­molecular hydrogen bonds involving O11—H11*D*⋯O1^ii^, O12^i^—H12*B* ^i^⋯O6 and O13^ii^—H13*A* ^ii^⋯O5 (Fig. 4 ▸ *a*); (II) inter­molecular hydrogen bonds involving O9—H9*B*⋯O6^i^ and equivalent O9^i^—H9*B* ^i^⋯O6 (Fig. 5 ▸ *a*) and (III) inter­molecular hydrogen bonds involving O12^i^—H12*A* ^i^⋯O8 and O14—H14*A*⋯O1^i^ (Fig. 6 ▸ *a*). The yellow zigzag chain connects with two neighbouring green chains *via* the group I inter­molecular hydrogen bonds, resulting in a two-dimensional supra­molecular layer (Fig. 4 ▸ *b*). The yellow chain also connects with the red and blue chains, assembling into two-dimensional supra­molecular layers *via* the inter­molecular hydrogen bonds of groups II (Fig. 5 ▸ *b*) and III (Fig. 6 ▸ *b*), respectively.

**Figure 7:**
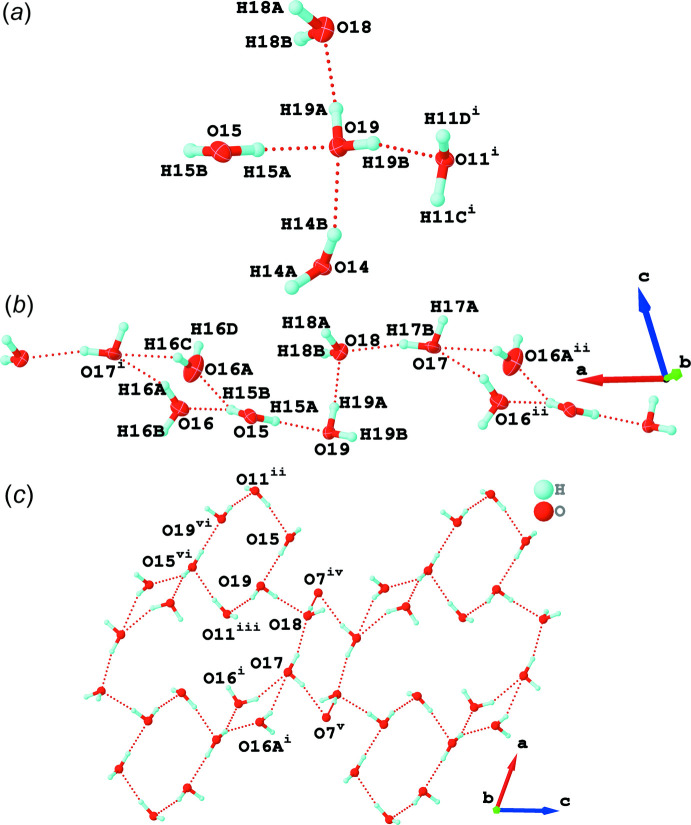
(*a*) A tetra­hedral water penta­mer with hydrogen bonds shown as red dashed lines [symmetry code: (i) 1 − *x*, 2 − *y*, 1 − *z*], (*b*) a one-dimensional water chain generated from the uncoordinated water mol­ecules [symmetry codes: (i) 1 + *x*, *y*, *z*; (ii) −1 + *x*, *y*, *z*] and (*c*) a one-dimensional water tape formed from hydrogen-bonded alternating six- and twelve-membered rings [symmetry codes: (i) −1 + *x*, *y*, *z*; (ii) 1 + *x*, *y*, *z*; (iii) 1 − *x*, 2 − *y*, 1 − *z*; (iv) 2 − *x*, 1 − *y*, 1 − *z*; (v) −1 + *x*, *y*, 1 + *z*; (vi) 2 − *x*, 2 − *y*, 1 − *z*].

In addition, there are a number of other hydrogen-bonding inter­actions within the structure. The free water mol­ecule H_2_O19 forms four hydrogen bonds, two with coordinated water mol­ecules H_2_O11 and H_2_O14, and two with free water mol­ecules H_2_O15 and H_2_O18 (Fig. 7 ▸ *a*), generating a tetra­hedral water penta­mer. Similar penta­mers have been observed previously (Saraei *et al.*, 2018 ▸; Liu *et al.*, 2020 ▸). In addition, the five free water mol­ecules H_2_O15, H_2_O16 (which is disordered over two positions, H_2_O16 and H_2_O16*A*), H_2_O17, H_2_O18 and H_2_O19 are linked into a one-dimensional water chain *via* hydrogen bonds (Fig. 7 ▸ *b*). The water chains are then further connected into a hydrogen-bonded supra­molecular layer *via* the coordinated water mol­ecule, H_2_O11, and the carboxyl­ate oxygen atom, O7 (Fig. 7 ▸ *c*). The resulting water layer contains alternating six- and twelve-membered oxygen rings and can be viewed as a one-dimensional *T*6 (3)12 (3) water tape. Similar water tapes have been reported previously (Han *et al.*, 2019 ▸; Liu *et al.*, 2012*a* ▸; Zhao *et al.*, 2019 ▸, Hao *et al.*, 2013 ▸).

## Database survey   

A survey of the Cambridge Structural Database (CSD version 5.42, May 2021 update; Groom *et al.*, 2016 ▸) reveals 19 structures containing H_4_adtp, three of which are Co^II^ complexes, including one two-dimensional coordination polymer (refcode CUFDIS; Ma *et al.*, 2021 ▸) and two discrete coordination complexes (RAXJUX and RAXKEI; Liu *et al.*, 2012*b* ▸). No structures containing H_4_adtp with similar cell parameters to those of the title compound have been reported.

## Synthesis and crystallization   

H_4_adtp was synthesized using a method based on that described in the literature (Xu *et al.*, 2006 ▸). The other chemicals were purchased from commercial sources and used without further purification. A mixture of Co(NO_3_)_2_·6H_2_O (0.2910 g, 1 mmol), H_4_adtp (0.0594 g, 0.2 mmol) and hexa­methyl­ene­tetra­mine (0.0701 g, 0.5 mmol) was dissolved in 6 mL of water. The solvent was allowed to evaporate slowly at room temperature. Crystals in the form of light-pink blocks were grown after one week, collected by filtration and dried in air. A 62% yield based on H_4_adtp was obtained. Analysis calculated (%) for C_12_H_29_N_1_O_19_Co_2_ (*M*_r_ = 609.22): C 23.66, H 4.80, N 2.30; found: C 23.66, H 4.77, N 2.33. Selected IR data (KBr pellet, cm^−1^): 3449 (*s*), 1901 (*w*), 1615 (*m*), 1568(*m*), 1386 (*m*), 1191 (*w*), 1092 (*w*), 787 (*w*), 733 (*w*).

The phase purity of (**I**) was demonstrated by powder X-ray diffraction analysis (PXRD; Fig. S1 in the supporting information). The peak positions of the experimental PXRD pattern match well with those simulated from the single-crystal X-ray data, indicating that the pure phase was synthesized.

## Refinement   

Crystal data, data collection and structure refinement details are summarized in Table 2 ▸. During the refinement of (**I**), O16 was found to be disordered over two sites (O16 and O16*A*) with occupancies of 0.704 (5) and 0.296 (5). The hydrogen atoms of the water mol­ecules were found in electron-density maps and refined as riding, with *U*_iso_(H) = 1.5 *U*_eq_(O). Other hydrogen atoms were placed at geometrically calculated positions and treated as riding, with C*sp* ^2^—H = 0.93 Å, C*sp* ^3^—H = 0.97 Å and *U*_iso_(H) = 1.2 *U*_eq_(C).

## Supplementary Material

Crystal structure: contains datablock(s) I. DOI: 10.1107/S2056989021008343/cq2045sup1.cif

Structure factors: contains datablock(s) I. DOI: 10.1107/S2056989021008343/cq2045Isup2.hkl

Supporting information file. DOI: 10.1107/S2056989021008343/cq2045Isup3.cdx

Figure S1. The simulated and experimental PXRD patterns for (I). DOI: 10.1107/S2056989021008343/cq2045sup4.tif

CCDC reference: 2063370

Additional supporting information:  crystallographic information; 3D view; checkCIF report

## Figures and Tables

**Table 1:** Hydrogen-bond geometry (Å, °)

*D*—H⋯*A*	*D*—H	H⋯*A*	*D*⋯*A*	*D*—H⋯*A*
O14—H14*A*⋯O1^i^	0.87	1.87	2.723 (2)	166
O14—H14*B*⋯O19	0.87	1.92	2.743 (2)	158
O9—H9*A*⋯O15^ii^	0.87	1.84	2.702 (2)	168
O9—H9*B*⋯O6^iii^	0.87	1.87	2.742 (2)	175
O13—H13*A*⋯O5^iv^	0.87	1.88	2.727 (2)	162
O13—H13*B*⋯O1^v^	0.87	1.98	2.759 (2)	148
O18—H18*A*⋯O5^vi^	0.87	1.93	2.752 (2)	158
O18—H18*B*⋯O7^i^	0.87	1.88	2.750 (2)	177
O15—H15*A*⋯O19	0.87	1.87	2.738 (2)	177
O15—H15*B*⋯O16	0.87	1.85	2.692 (3)	162
O15—H15*B*⋯O16*A*	0.87	2.02	2.833 (7)	156
O11—H11*C*⋯O15^vii^	0.87	1.82	2.686 (2)	172
O11—H11*D*⋯O1^iv^	0.87	1.95	2.786 (2)	161
O10—H10*A*⋯O17^viii^	0.87	1.88	2.705 (2)	158
O10—H10*B*⋯O4	0.87	2.11	2.740 (2)	129
O12—H12*A*⋯O8^i^	0.87	1.92	2.773 (2)	166
O12—H12*B*⋯O6^ix^	0.87	1.98	2.846 (2)	172
O19—H19*A*⋯O18	0.87	1.85	2.719 (2)	175
O19—H19*B*⋯O11^viii^	0.87	1.94	2.786 (2)	165
O17—H17*A*⋯O7^x^	0.87	1.88	2.702 (2)	157
O17—H17*B*⋯O18	0.87	1.95	2.804 (2)	166
O16*A*—H16*C*⋯O17^xi^	0.87	2.11	2.861 (8)	144
O16—H16*A*⋯O17^xi^	0.87	2.10	2.921 (4)	156

Symmetry codes: (i) -x+2, -y+1, -z+1; (ii) -x+2, -y+2, -z+1; (iii) -x+2, -y+2, -z; (iv) -x+1, -y+1, -z+1; (v) -x+1, -y, -z+2; (vi) x, y, z+1; (vii) x-1, y, z; (viii) -x+1, -y+2, -z+1; (ix) x, y-1, z+1; (x) x-1, y, z+1; (xi) x+1, y, z.

**Table 2:** Experimental details

Crystal data	
Chemical formula	[Co_2_(C_12_H_7_NO_8_)(H_2_O)_6_]·5H_2_O
*M*_r_	609.22
Crystal system, space group	Triclinic, *P*\overline{1}
Temperature (K)	293
*a*, *b*, *c* (Å)	9.7653 (15), 11.725 (2), 11.8191 (15)
α, β, γ (°)	64.882 (5), 71.276 (7), 86.692 (8)
*V* (Å^3^)	1155.6 (3)
*Z*	2
Radiation type	Mo *K*α
μ (mm^−1^)	1.53
Crystal size (mm)	0.2 × 0.2 × 0.2

Data collection	
Diffractometer	Rigaku Saturn724+ (2x2 bin mode)
Absorption correction	Multi-scan (*CrystalClear*; Rigaku, 2008 ▸)
*T*_min_, *T*_max_	0.844, 1.000
No. of measured, independent and observed [*I* > 2σ(*I*)] reflections	10193, 4052, 3498
*R*_int_	0.029
(sin θ/λ)_max_ (Å^−1^)	0.595

Refinement	
*R*[*F*^2^ > 2σ(*F* ^2^)], *wR*(*F* ^2^), *S*	0.027, 0.069, 1.02
No. of reflections	4052
No. of parameters	348
No. of restraints	2
H-atom treatment	H-atom parameters constrained
Δρ_max_, Δρ_min_ (e Å^−3^)	0.97, −0.45

Computer programs: *CrystalClear* (Rigaku, 2008 ▸), *SHELXS* (Sheldrick, 2008 ▸), *SHELXL2018/3* (Sheldrick, 2015 ▸) and *OLEX2* (Dolomanov *et al.*, 2009 ▸).

## supplementary crystallographic information

### Crystal data

**Table:**

[Co_2_(C_12_H_7_NO_8_)(H_2_O)_6_]·5H_2_O	*Z* = 2
*M**_r_* = 609.22	*F*(000) = 628
Triclinic, *P*1	*D*_x_ = 1.751 Mg m^−^^3^
*a* = 9.7653 (15) Å	Mo *K*α radiation, λ = 0.71073 Å
*b* = 11.725 (2) Å	Cell parameters from 3650 reflections
*c* = 11.8191 (15) Å	θ = 2.0–27.5°
α = 64.882 (5)°	µ = 1.53 mm^−^^1^
β = 71.276 (7)°	*T* = 293 K
γ = 86.692 (8)°	Block, clear light red
*V* = 1155.6 (3) Å^3^	0.2 × 0.2 × 0.2 mm

### Data collection

**Table:**

Rigaku Saturn724+ (2x2 bin mode) diffractometer	4052 independent reflections
Radiation source: Sealed Tube, Rotating Anode	3498 reflections with *I* > 2σ(*I*)
Graphite monochromator	*R*_int_ = 0.029
Detector resolution: 28.5714 pixels mm^-1^	θ_max_ = 25.0°, θ_min_ = 2.1°
CCD_Profile_fitting scans	*h* = −11→11
Absorption correction: multi-scan (CrystalClear; Rigaku, 2008)	*k* = −13→13
*T*_min_ = 0.844, *T*_max_ = 1.000	*l* = −14→14
10193 measured reflections	

### Refinement

**Table:**

Refinement on *F*^2^	Primary atom site location: structure-invariant direct methods
Least-squares matrix: full	Hydrogen site location: mixed
*R*[*F*^2^ > 2σ(*F*^2^)] = 0.027	H-atom parameters constrained
*wR*(*F*^2^) = 0.069	*w* = 1/[σ^2^(*F*_o_^2^) + (0.0373*P*)^2^] where *P* = (*F*_o_^2^ + 2*F*_c_^2^)/3
*S* = 1.02	(Δ/σ)_max_ < 0.001
4052 reflections	Δρ_max_ = 0.97 e Å^−^^3^
348 parameters	Δρ_min_ = −0.45 e Å^−^^3^
2 restraints	

### Special details

**Table:**

Geometry. All esds (except the esd in the dihedral angle between two l.s. planes) are estimated using the full covariance matrix. The cell esds are taken into account individually in the estimation of esds in distances, angles and torsion angles; correlations between esds in cell parameters are only used when they are defined by crystal symmetry. An approximate (isotropic) treatment of cell esds is used for estimating esds involving l.s. planes.

### Fractional atomic coordinates and isotropic or equivalent isotropic displacement parameters (Å^2^)

**Table:**

	*x*	*y*	*z*	*U*_iso_*/*U*_eq_	Occ. (<1)
Co1	0.500000	0.000000	1.000000	0.01013 (11)	
O1	0.71506 (15)	0.16282 (13)	0.67122 (14)	0.0116 (3)	
C1	0.6534 (2)	0.36754 (19)	0.6586 (2)	0.0097 (4)	
Co2	0.92914 (3)	0.81788 (3)	0.25427 (3)	0.00926 (9)	
O2	0.53599 (15)	0.17746 (13)	0.83918 (14)	0.0128 (3)	
C2	0.7374 (2)	0.43193 (19)	0.5263 (2)	0.0097 (4)	
H2	0.787693	0.385882	0.481028	0.012*	
Co3	0.500000	1.000000	0.500000	0.00977 (11)	
O3	0.53886 (16)	0.81035 (13)	0.52932 (14)	0.0142 (3)	
O14	0.99581 (16)	0.76339 (14)	0.42109 (14)	0.0152 (3)	
H14A	1.084005	0.796585	0.396511	0.023*	
H14B	0.943474	0.796773	0.472202	0.023*	
C3	0.7484 (2)	0.56273 (19)	0.4598 (2)	0.0090 (4)	
O4	0.72895 (16)	0.84545 (13)	0.35178 (15)	0.0158 (3)	
O9	1.00200 (19)	1.00342 (14)	0.17472 (15)	0.0207 (4)	
H9A	0.971354	1.053917	0.212391	0.031*	
H9B	1.039011	1.053478	0.090961	0.031*	
C4	0.6636 (2)	0.63255 (19)	0.5254 (2)	0.0098 (4)	
O5	0.78350 (17)	0.73838 (14)	0.00522 (15)	0.0163 (3)	
C5	0.5863 (2)	0.5667 (2)	0.6599 (2)	0.0113 (5)	
H5	0.535647	0.611872	0.706117	0.014*	
O6	0.86627 (16)	0.84612 (13)	0.08939 (14)	0.0128 (3)	
C6	0.5822 (2)	0.43705 (19)	0.7268 (2)	0.0111 (5)	
H6	0.532011	0.396404	0.817033	0.013*	
O7	1.19306 (16)	0.59855 (14)	0.11284 (15)	0.0188 (4)	
O13	0.36628 (16)	0.07573 (13)	1.12298 (15)	0.0137 (3)	
H13A	0.305886	0.121648	1.085472	0.021*	
H13B	0.310559	0.014679	1.194882	0.021*	
C7	0.6342 (2)	0.22445 (19)	0.7282 (2)	0.0102 (5)	
O18	0.69274 (18)	0.62405 (15)	0.87758 (17)	0.0216 (4)	
H18A	0.722316	0.639556	0.932159	0.032*	
H18B	0.730870	0.554995	0.878060	0.032*	
O8	1.11592 (15)	0.75971 (13)	0.16099 (14)	0.0126 (3)	
C8	0.6440 (2)	0.77274 (19)	0.4638 (2)	0.0099 (5)	
C9	0.9740 (2)	0.55842 (19)	0.2918 (2)	0.0123 (5)	
H9C	0.950428	0.492589	0.270057	0.015*	
H9D	1.001629	0.517939	0.370768	0.015*	
C10	1.1036 (2)	0.6459 (2)	0.1777 (2)	0.0108 (5)	
O15	1.07588 (17)	0.85972 (15)	0.68317 (17)	0.0201 (4)	
H15A	0.999917	0.839996	0.669056	0.030*	
H15B	1.116676	0.789336	0.707557	0.030*	
C11	0.7564 (2)	0.6353 (2)	0.2337 (2)	0.0124 (5)	
H11A	0.655783	0.642055	0.277716	0.015*	
H11B	0.760380	0.557885	0.222209	0.015*	
C12	0.8078 (2)	0.74643 (19)	0.0985 (2)	0.0113 (5)	
O11	0.30500 (16)	0.97725 (13)	0.46413 (15)	0.0140 (3)	
H11C	0.236723	0.937054	0.539337	0.021*	
H11D	0.317123	0.927325	0.425117	0.021*	
O10	0.60855 (18)	1.07131 (14)	0.30338 (15)	0.0183 (4)	
H10A	0.585872	1.133580	0.240798	0.027*	
H10B	0.655576	1.024557	0.265690	0.027*	
N1	0.84205 (18)	0.62558 (16)	0.32063 (17)	0.0089 (4)	
O12	0.68118 (15)	0.04542 (14)	1.04035 (14)	0.0140 (3)	
H12A	0.733414	0.111214	0.971905	0.021*	
H12B	0.738442	−0.015215	1.048118	0.021*	
O19	0.83677 (17)	0.80778 (15)	0.63399 (16)	0.0190 (4)	
H19A	0.786359	0.749532	0.710713	0.028*	
H19B	0.779773	0.868110	0.616878	0.028*	
O17	0.41401 (19)	0.69892 (17)	0.88122 (16)	0.0286 (4)	
H17A	0.353610	0.681177	0.960127	0.043*	
H17B	0.496366	0.676069	0.893476	0.043*	
O16	1.2542 (3)	0.6732 (2)	0.7213 (3)	0.0262 (8)	0.704 (5)
H16A	1.279312	0.667157	0.787902	0.039*	0.704 (5)
H16B	1.332880	0.705061	0.653351	0.039*	0.704 (5)
O16A	1.1659 (10)	0.6285 (7)	0.8383 (9)	0.048 (3)	0.296 (5)
H16C	1.210697	0.664527	0.869032	0.071*	0.296 (5)
H16D	1.117075	0.561697	0.907255	0.071*	0.296 (5)

### Atomic displacement parameters (Å^2^)

**Table:**

	*U*^11^	*U*^22^	*U*^33^	*U*^12^	*U*^13^	*U*^23^
Co1	0.0094 (2)	0.0072 (2)	0.0088 (2)	−0.00013 (16)	0.00000 (18)	−0.00093 (17)
O1	0.0127 (8)	0.0080 (7)	0.0114 (8)	0.0007 (6)	−0.0014 (7)	−0.0037 (6)
C1	0.0082 (11)	0.0080 (11)	0.0119 (11)	0.0000 (8)	−0.0045 (9)	−0.0024 (9)
Co2	0.00941 (17)	0.00736 (16)	0.00848 (16)	−0.00012 (12)	−0.00072 (13)	−0.00260 (12)
O2	0.0119 (8)	0.0074 (8)	0.0098 (8)	−0.0006 (6)	0.0021 (7)	0.0008 (6)
C2	0.0097 (11)	0.0101 (11)	0.0098 (11)	0.0021 (9)	−0.0027 (9)	−0.0054 (9)
Co3	0.0105 (2)	0.0074 (2)	0.0096 (2)	0.00172 (16)	−0.00197 (18)	−0.00311 (17)
O3	0.0135 (8)	0.0097 (8)	0.0136 (8)	0.0030 (6)	0.0013 (7)	−0.0043 (7)
O14	0.0111 (8)	0.0197 (8)	0.0139 (8)	−0.0006 (7)	−0.0018 (7)	−0.0079 (7)
C3	0.0073 (11)	0.0099 (11)	0.0080 (11)	−0.0009 (8)	−0.0019 (9)	−0.0024 (9)
O4	0.0135 (8)	0.0086 (8)	0.0137 (8)	0.0024 (6)	0.0030 (7)	0.0000 (7)
O9	0.0336 (10)	0.0086 (8)	0.0113 (8)	−0.0064 (7)	0.0039 (8)	−0.0036 (7)
C4	0.0090 (11)	0.0085 (11)	0.0113 (11)	−0.0008 (8)	−0.0038 (9)	−0.0032 (9)
O5	0.0231 (9)	0.0142 (8)	0.0133 (8)	0.0020 (7)	−0.0090 (7)	−0.0054 (7)
C5	0.0094 (11)	0.0120 (11)	0.0138 (11)	0.0011 (9)	−0.0025 (9)	−0.0076 (9)
O6	0.0163 (8)	0.0089 (8)	0.0110 (8)	−0.0011 (6)	−0.0045 (7)	−0.0020 (6)
C6	0.0094 (11)	0.0124 (11)	0.0075 (11)	−0.0025 (9)	−0.0005 (9)	−0.0018 (9)
O7	0.0154 (9)	0.0161 (8)	0.0184 (9)	0.0019 (7)	0.0033 (7)	−0.0080 (7)
O13	0.0148 (8)	0.0093 (8)	0.0117 (8)	0.0009 (6)	−0.0022 (7)	−0.0012 (6)
C7	0.0093 (11)	0.0090 (11)	0.0121 (11)	0.0009 (9)	−0.0064 (10)	−0.0022 (9)
O18	0.0274 (10)	0.0159 (9)	0.0283 (10)	0.0056 (7)	−0.0134 (8)	−0.0131 (8)
O8	0.0103 (8)	0.0100 (8)	0.0133 (8)	−0.0001 (6)	0.0001 (7)	−0.0040 (6)
C8	0.0101 (11)	0.0104 (11)	0.0122 (11)	0.0011 (9)	−0.0056 (10)	−0.0061 (9)
C9	0.0126 (12)	0.0105 (11)	0.0120 (11)	0.0024 (9)	−0.0023 (10)	−0.0045 (9)
C10	0.0095 (11)	0.0119 (11)	0.0104 (11)	0.0029 (9)	−0.0051 (9)	−0.0032 (9)
O15	0.0164 (9)	0.0221 (9)	0.0240 (9)	−0.0002 (7)	−0.0039 (8)	−0.0138 (8)
C11	0.0104 (11)	0.0131 (11)	0.0127 (11)	−0.0010 (9)	−0.0031 (10)	−0.0049 (9)
C12	0.0073 (11)	0.0130 (12)	0.0136 (12)	0.0051 (9)	−0.0039 (9)	−0.0060 (9)
O11	0.0135 (8)	0.0141 (8)	0.0146 (8)	0.0006 (6)	−0.0027 (7)	−0.0078 (7)
O10	0.0257 (10)	0.0135 (8)	0.0118 (8)	0.0076 (7)	−0.0017 (7)	−0.0058 (7)
N1	0.0080 (9)	0.0086 (9)	0.0061 (9)	0.0007 (7)	−0.0001 (8)	−0.0012 (7)
O12	0.0116 (8)	0.0103 (8)	0.0139 (8)	0.0001 (6)	−0.0009 (7)	−0.0019 (7)
O19	0.0179 (9)	0.0206 (9)	0.0185 (9)	0.0059 (7)	−0.0065 (8)	−0.0087 (7)
O17	0.0254 (10)	0.0326 (11)	0.0146 (9)	0.0077 (8)	−0.0009 (8)	−0.0030 (8)
O16	0.0289 (16)	0.0194 (14)	0.0251 (18)	−0.0011 (12)	−0.0017 (13)	−0.0095 (13)
O16A	0.059 (6)	0.033 (4)	0.074 (7)	0.018 (4)	−0.044 (6)	−0.029 (4)

### Geometric parameters (Å, º)

**Table:**

Co1—O2^i^	2.0890 (14)	O5—C12	1.240 (3)
Co1—O2	2.0890 (14)	C5—H5	0.9300
Co1—O13^i^	2.0856 (15)	C5—C6	1.380 (3)
Co1—O13	2.0856 (15)	O6—C12	1.279 (3)
Co1—O12^i^	2.1231 (15)	C6—H6	0.9300
Co1—O12	2.1231 (15)	O7—C10	1.239 (3)
O1—C7	1.267 (3)	O13—H13A	0.8734
C1—C2	1.391 (3)	O13—H13B	0.8731
C1—C6	1.389 (3)	O18—H18A	0.8706
C1—C7	1.514 (3)	O18—H18B	0.8700
Co2—O14	2.1039 (15)	O8—C10	1.270 (3)
Co2—O4	2.0128 (15)	C9—H9C	0.9700
Co2—O9	2.0336 (15)	C9—H9D	0.9700
Co2—O6	2.1168 (15)	C9—C10	1.534 (3)
Co2—O8	2.0521 (15)	C9—N1	1.492 (3)
Co2—N1	2.1712 (18)	O15—H15A	0.8738
O2—C7	1.259 (2)	O15—H15B	0.8699
C2—H2	0.9300	C11—H11A	0.9700
C2—C3	1.387 (3)	C11—H11B	0.9700
Co3—O3	2.1311 (14)	C11—C12	1.516 (3)
Co3—O3^ii^	2.1311 (14)	C11—N1	1.486 (3)
Co3—O11	2.1330 (14)	O11—H11C	0.8700
Co3—O11^ii^	2.1330 (15)	O11—H11D	0.8692
Co3—O10^ii^	2.0234 (15)	O10—H10A	0.8702
Co3—O10	2.0235 (15)	O10—H10B	0.8699
O3—C8	1.255 (3)	O12—H12A	0.8720
O14—H14A	0.8716	O12—H12B	0.8707
O14—H14B	0.8710	O19—H19A	0.8703
C3—C4	1.416 (3)	O19—H19B	0.8698
C3—N1	1.472 (3)	O17—H17A	0.8702
O4—C8	1.260 (2)	O17—H17B	0.8697
O9—H9A	0.8696	O16—H16A	0.8710
O9—H9B	0.8699	O16—H16B	0.8700
C4—C5	1.395 (3)	O16A—H16C	0.8695
C4—C8	1.518 (3)	O16A—H16D	0.8699
			
O2^i^—Co1—O2	180.0	Co2—O9—H9B	126.4
O2^i^—Co1—O12^i^	89.84 (6)	H9A—O9—H9B	104.6
O2—Co1—O12^i^	90.16 (6)	C3—C4—C8	126.71 (19)
O2—Co1—O12	89.84 (6)	C5—C4—C3	117.58 (18)
O2^i^—Co1—O12	90.16 (6)	C5—C4—C8	115.68 (18)
O13—Co1—O2	89.80 (6)	C4—C5—H5	118.8
O13^i^—Co1—O2^i^	89.80 (6)	C6—C5—C4	122.4 (2)
O13—Co1—O2^i^	90.20 (6)	C6—C5—H5	118.8
O13^i^—Co1—O2	90.20 (6)	C12—O6—Co2	114.05 (13)
O13^i^—Co1—O13	180.0	C1—C6—H6	120.2
O13^i^—Co1—O12^i^	89.48 (6)	C5—C6—C1	119.66 (19)
O13—Co1—O12^i^	90.52 (6)	C5—C6—H6	120.2
O13—Co1—O12	89.48 (6)	Co1—O13—H13A	109.5
O13^i^—Co1—O12	90.52 (6)	Co1—O13—H13B	109.4
O12—Co1—O12^i^	180.0	H13A—O13—H13B	104.3
C2—C1—C7	121.71 (19)	O1—C7—C1	118.59 (18)
C6—C1—C2	118.73 (19)	O2—C7—O1	125.81 (19)
C6—C1—C7	119.54 (19)	O2—C7—C1	115.60 (18)
O14—Co2—O6	171.86 (6)	H18A—O18—H18B	104.5
O14—Co2—N1	91.18 (6)	C10—O8—Co2	114.07 (13)
O4—Co2—O14	92.18 (6)	O3—C8—O4	122.76 (19)
O4—Co2—O9	93.01 (7)	O3—C8—C4	116.55 (18)
O4—Co2—O6	91.31 (6)	O4—C8—C4	120.68 (18)
O4—Co2—O8	169.72 (6)	H9C—C9—H9D	107.7
O4—Co2—N1	86.41 (6)	C10—C9—H9C	108.9
O9—Co2—O14	95.30 (6)	C10—C9—H9D	108.9
O9—Co2—O6	91.86 (6)	N1—C9—H9C	108.9
O9—Co2—O8	96.83 (6)	N1—C9—H9D	108.9
O9—Co2—N1	173.51 (7)	N1—C9—C10	113.37 (16)
O6—Co2—N1	81.70 (6)	O7—C10—O8	124.6 (2)
O8—Co2—O14	89.88 (6)	O7—C10—C9	117.42 (18)
O8—Co2—O6	85.42 (6)	O8—C10—C9	117.84 (18)
O8—Co2—N1	83.48 (6)	H15A—O15—H15B	103.9
C7—O2—Co1	132.21 (13)	H11A—C11—H11B	107.6
C1—C2—H2	119.0	C12—C11—H11A	108.7
C3—C2—C1	121.99 (19)	C12—C11—H11B	108.7
C3—C2—H2	119.0	N1—C11—H11A	108.7
O3—Co3—O3^ii^	180.0	N1—C11—H11B	108.7
O3^ii^—Co3—O11	90.75 (6)	N1—C11—C12	114.38 (17)
O3^ii^—Co3—O11^ii^	89.25 (6)	O5—C12—O6	123.83 (19)
O3—Co3—O11	89.25 (6)	O5—C12—C11	118.53 (19)
O3—Co3—O11^ii^	90.75 (6)	O6—C12—C11	117.50 (18)
O11—Co3—O11^ii^	180.00 (8)	Co3—O11—H11C	109.3
O10—Co3—O3	93.04 (6)	Co3—O11—H11D	109.3
O10—Co3—O3^ii^	86.96 (6)	H11C—O11—H11D	104.5
O10^ii^—Co3—O3^ii^	93.04 (6)	Co3—O10—H10A	127.0
O10^ii^—Co3—O3	86.96 (6)	Co3—O10—H10B	122.3
O10^ii^—Co3—O11	90.32 (6)	H10A—O10—H10B	104.5
O10^ii^—Co3—O11^ii^	89.68 (6)	C3—N1—Co2	115.69 (12)
O10—Co3—O11	89.68 (6)	C3—N1—C9	112.36 (16)
O10—Co3—O11^ii^	90.32 (6)	C3—N1—C11	109.16 (16)
O10^ii^—Co3—O10	180.0	C9—N1—Co2	103.66 (12)
C8—O3—Co3	127.99 (13)	C11—N1—Co2	105.14 (12)
Co2—O14—H14A	109.5	C11—N1—C9	110.49 (16)
Co2—O14—H14B	109.4	Co1—O12—H12A	109.4
H14A—O14—H14B	104.4	Co1—O12—H12B	109.4
C2—C3—C4	119.14 (19)	H12A—O12—H12B	104.5
C2—C3—N1	119.40 (18)	H19A—O19—H19B	104.5
C4—C3—N1	121.39 (18)	H17A—O17—H17B	104.5
C8—O4—Co2	128.55 (13)	H16A—O16—H16B	104.4
Co2—O9—H9A	124.8	H16C—O16A—H16D	104.6
			
Co1—O2—C7—O1	21.8 (3)	C4—C3—N1—C9	148.70 (19)
Co1—O2—C7—C1	−158.96 (13)	C4—C3—N1—C11	−88.4 (2)
C1—C2—C3—C4	−4.6 (3)	C4—C5—C6—C1	−2.0 (3)
C1—C2—C3—N1	178.43 (18)	C5—C4—C8—O3	−15.1 (3)
Co2—O4—C8—O3	161.71 (15)	C5—C4—C8—O4	165.91 (19)
Co2—O4—C8—C4	−19.4 (3)	C6—C1—C2—C3	−2.0 (3)
Co2—O6—C12—O5	−168.82 (16)	C6—C1—C7—O1	−170.27 (19)
Co2—O6—C12—C11	15.5 (2)	C6—C1—C7—O2	10.5 (3)
Co2—O8—C10—O7	166.73 (17)	C7—C1—C2—C3	176.06 (19)
Co2—O8—C10—C9	−17.3 (2)	C7—C1—C6—C5	−172.81 (18)
C2—C1—C6—C5	5.3 (3)	C8—C4—C5—C6	173.80 (19)
C2—C1—C7—O1	11.7 (3)	C10—C9—N1—Co2	−26.61 (19)
C2—C1—C7—O2	−167.56 (19)	C10—C9—N1—C3	−152.23 (17)
C2—C3—C4—C5	7.8 (3)	C10—C9—N1—C11	85.6 (2)
C2—C3—C4—C8	−170.45 (19)	C12—C11—N1—Co2	27.1 (2)
C2—C3—N1—Co2	−153.20 (15)	C12—C11—N1—C3	151.78 (17)
C2—C3—N1—C9	−34.4 (3)	C12—C11—N1—C9	−84.2 (2)
C2—C3—N1—C11	88.5 (2)	N1—C3—C4—C5	−175.36 (18)
Co3—O3—C8—O4	−15.4 (3)	N1—C3—C4—C8	6.4 (3)
Co3—O3—C8—C4	165.66 (13)	N1—C9—C10—O7	−152.01 (19)
C3—C4—C5—C6	−4.6 (3)	N1—C9—C10—O8	31.7 (3)
C3—C4—C8—O3	163.1 (2)	N1—C11—C12—O5	153.61 (19)
C3—C4—C8—O4	−15.9 (3)	N1—C11—C12—O6	−30.4 (3)
C4—C3—N1—Co2	29.9 (2)		

Symmetry codes: (i) −*x*+1, −*y*, −*z*+2; (ii) −*x*+1, −*y*+2, −*z*+1.

### Hydrogen-bond geometry (Å, º)

**Table:**

*D*—H···*A*	*D*—H	H···*A*	*D*···*A*	*D*—H···*A*
O14—H14*A*···O1^iii^	0.87	1.87	2.723 (2)	166
O14—H14*B*···O19	0.87	1.92	2.743 (2)	158
O9—H9*A*···O15^iv^	0.87	1.84	2.702 (2)	168
O9—H9*B*···O6^v^	0.87	1.87	2.742 (2)	175
O13—H13*A*···O5^vi^	0.87	1.88	2.727 (2)	162
O13—H13*B*···O1^i^	0.87	1.98	2.759 (2)	148
O18—H18*A*···O5^vii^	0.87	1.93	2.752 (2)	158
O18—H18*B*···O7^iii^	0.87	1.88	2.750 (2)	177
O15—H15*A*···O19	0.87	1.87	2.738 (2)	177
O15—H15*B*···O16	0.87	1.85	2.692 (3)	162
O15—H15*B*···O16*A*	0.87	2.02	2.833 (7)	156
O11—H11*C*···O15^viii^	0.87	1.82	2.686 (2)	172
O11—H11*D*···O1^vi^	0.87	1.95	2.786 (2)	161
O10—H10*A*···O17^ii^	0.87	1.88	2.705 (2)	158
O10—H10*B*···O4	0.87	2.11	2.740 (2)	129
O12—H12*A*···O8^iii^	0.87	1.92	2.773 (2)	166
O12—H12*B*···O6^ix^	0.87	1.98	2.846 (2)	172
O19—H19*A*···O18	0.87	1.85	2.719 (2)	175
O19—H19*B*···O11^ii^	0.87	1.94	2.786 (2)	165
O17—H17*A*···O7^x^	0.87	1.88	2.702 (2)	157
O17—H17*B*···O18	0.87	1.95	2.804 (2)	166
O16*A*—H16*C*···O17^xi^	0.87	2.11	2.861 (8)	144
O16—H16*A*···O17^xi^	0.87	2.10	2.921 (4)	156

Symmetry codes: (i) −*x*+1, −*y*, −*z*+2; (ii) −*x*+1, −*y*+2, −*z*+1; (iii) −*x*+2, −*y*+1, −*z*+1; (iv) −*x*+2, −*y*+2, −*z*+1; (v) −*x*+2, −*y*+2, −*z*; (vi) −*x*+1, −*y*+1, −*z*+1; (vii) *x*, *y*, *z*+1; (viii) *x*−1, *y*, *z*; (ix) *x*, *y*−1, *z*+1; (x) *x*−1, *y*, *z*+1; (xi) *x*+1, *y*, *z*.
